# An efficient method to find potentially universal population genetic markers, applied to metazoans

**DOI:** 10.1186/1471-2148-10-276

**Published:** 2010-09-13

**Authors:** Anne Chenuil, Thierry B Hoareau, Emilie Egea, Gwilherm Penant, Caroline Rocher, Didier Aurelle, Kenza Mokhtar-Jamai, John DD Bishop, Emilie Boissin, Angie Diaz, Manuela Krakau, Pieternella C Luttikhuizen, Francesco P Patti, Nicolas Blavet, Sylvain Mousset

**Affiliations:** 1 African Coelacanth Ecosystem Programme, Department of Genetics, University of Pretoria, Lynnwood Road 0002, Pretoria, South Africa; 2 Marine Biological Association of the UK, The Laboratory, Citadel Hill Plymouth, PL1 2PB, UK; 3 Department of Genetics, Forestry and Agricultural Biotechnology Institute (FABI), University of Pretoria, Pretoria, South Africa; 4 Instituto de Ecología y Biodiversidad, Departamento de Ciencias Ecológicas, Facultad de Ciencias, Universidad de Chile, Santiago, Chile; 5 Alfred Wegener Institute for Polar and Marine Research, Wadden Sea, Station Sylt, Hafenstr. 43, 25992 List, Germany; 6 Royal Netherlands Institute for Sea Research, Department of Marine Ecology, P.O. Box 59, 1790AB Den Burg, The Netherlands; 7 Functional and Evolutionary Ecology Laboratory, Stazione Zoologica "Anton Dohrn", Villa Dohrn, Punta S. Pietro, Napoli, Italia - 80077 Ischia (Naples), Italy; 8 Plant Ecological Genetics Group, Institute of Integrative Biology, ETH Zurich, Universitätsstrasse 16, CHN, 8092 Zurich, Switzerland; 9 Université de Lyon, F-69000, Lyon, France; 10 Université Lyon 1; CNRS, UMR5558, Laboratoire de Biométrie et Biologie Evolutive, F-69622, Villeurbanne, France; 11 Aix-Marseille Université, Laboratoire DIMAR (diversité, évolution et écologie fonctionnelle marine), CNRS UMR6540, rue de la batterie des Lions, 13007 Marseille, France

## Abstract

**Background:**

Despite the impressive growth of sequence databases, the limited availability of nuclear markers that are sufficiently polymorphic for population genetics and phylogeography and applicable across various phyla restricts many potential studies, particularly in non-model organisms. Numerous introns have invariant positions among kingdoms, providing a potential source for such markers. Unfortunately, most of the few known EPIC (Exon Primed Intron Crossing) loci are restricted to vertebrates or belong to multigenic families.

**Results:**

In order to develop markers with broad applicability, we designed a bioinformatic approach aimed at avoiding multigenic families while identifying intron positions conserved across metazoan phyla. We developed a program facilitating the identification of EPIC loci which allowed slight variation in intron position. From the *Homolens *databases we selected 29 gene families which contained 52 promising introns for which we designed 93 primer pairs. PCR tests were performed on several ascidians, echinoderms, bivalves and cnidarians. On average, 24 different introns per genus were amplified in bilaterians. Remarkably, five of the introns successfully amplified in all of the metazoan genera tested (a dozen genera, including cnidarians). The influence of several factors on amplification success was investigated. Success rate was not related to the phylogenetic relatedness of a taxon to the groups that most influenced primer design, showing that these EPIC markers are extremely conserved in animals.

**Conclusions:**

Our new method now makes it possible to (i) rapidly isolate a set of EPIC markers for any phylum, even outside the animal kingdom, and thus, (ii) compare genetic diversity at potentially homologous polymorphic loci between divergent taxa.

## Background

Despite the crucial need for genetic markers independent from the non recombining mitochondrial genome, nuclear markers remain much less used than mitochondrial ones in metazoans (in the Entrez-Nucleotide database, there are about ten times fewer entries containing "population" and "nuclear marker" or "population" and "nuclear", when used as key-words, than entries containing "mitochondrial" instead of "nuclear"). When choosing molecular genetic markers for a given biodiversity study, two properties, codominance and the possibility of reconstructing evolutionary relationships among alleles, are generally desirable but are often difficult to obtain [1]. During the last decade, microsatellites became the most popular codominant markers. However, introns are well known for providing potential markers variable within species, using EPIC-PCR. EPIC loci have several advantages compared to microsatellites. Owing to the position of the primers in conserved exons, EPICs are potentially applicable across species and much less prone to null alleles. After sequencing the variants, evolutionary relationships among alleles can be inferred much more accurately than for microsatellites, which are very susceptible to homoplasy [2]. There is also a less well known but important problem with microsatellites: in some species, most microsatellites appear to belong to the same family (or a small number of families) of repeated elements; in such cases codominant genotyping is difficult since the primers often anneal to multiple paralogous regions ([3,4] and Chenuil, unpublished).

A recent computer program [5] allows the identification of introns at conserved positions in a species for which EST sequences are available by comparison with a related model species for which extensive sequence information is available (see also [6]). Another study developed a bioinformatic pipeline to identify EPIC loci by comparison of two or more whole-genome sequence datasets, and tested a dozen of these loci by PCR-sequencing in distantly related teleost fishes [7]. By contrast, our approach is designed to find intron positions and define primers able to amplify a wider variety of species from which we may have absolutely no sequence data.

The positions of introns are extremely conserved during evolution; for instance, 14% of animal introns match plant intron positions [8,9]. Although this should have favoured the development of EPIC markers conserved in different phyla, only half a dozen EPIC loci were proposed by [10] and by [11]. These loci have rarely been used outside vertebrates (e.g. [12-14]) and in numerous species none of the tested EPIC loci appeared usable (e.g. [15]). EPIC loci can be developed specifically for a given taxon, often using genomic and cDNA sequence data (e.g. [16,17]; Aurelle *et al*., submitted). Only vertebrates benefit from a relatively consistent set of EPIC loci [18,19]. Several reasons for such biases can be invoked. (1) These "universal" EPIC loci were chosen in extremely conserved genes, and, not surprisingly, often appeared to belong to multigenic families, which limited their use as codominant markers due to simultaneous amplification of paralogs. (2) Few sequences were available (often none outside vertebrates) to properly define PCR primers, thus PCR amplifications often failed. (3) Primers were generally designed considering the nucleotide variation observed in the data set available (often phylogenetically limited), but ignoring amino-acid conservation and code degeneracy.

Our study was designed to avoid these shortcomings. We identified putative universal EPIC loci and designed primers to amplify them in metazoans, taking advantage of the increased availability of properly annotated, phylogenetically diverse whole-genome sequences. We actively avoided multigenic families, and used a primer design strategy aimed at preserving amino-acid sequences while allowing synonymous codon changes. For this purpose, we developed dedicated bioinformatic tools, and then tested all the primer pairs designed in divergent animal phyla for PCR amplification under relatively standardized conditions.

## Results-Discussion

### Potential EPIC loci found in data bases

**Figure 1:**
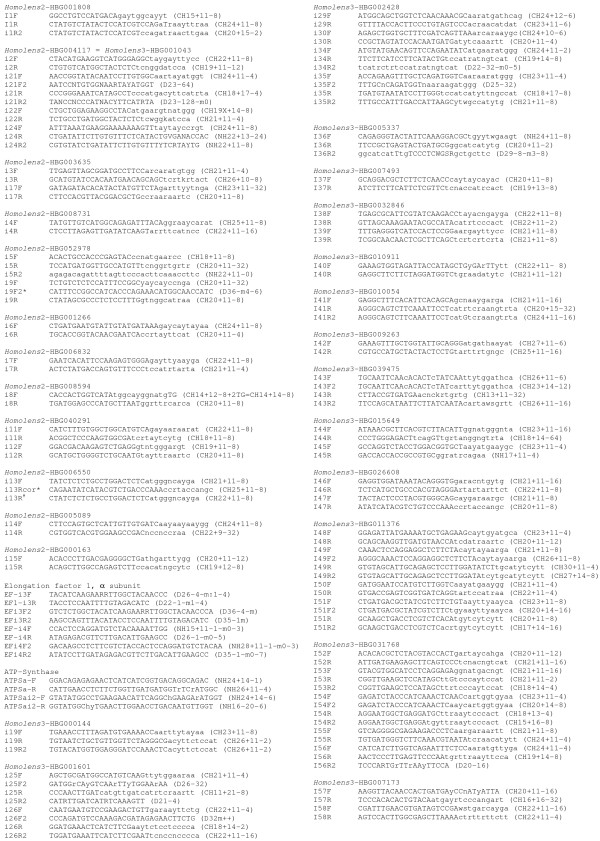
**Primer sequences**. Sequences are written using the IUPAC code. We used the following codes to describe the rules that underpinned primer design: Capital letters represent the 5'clamp (non-degenerate), whereas small letters represent the degenerate part supposed to contain no mismatch whatever the species (based on known sequences, protein for CH primers or nucleotide for D or NH primers). CHX+Y-z: CodeHop primer with a 5'clamp (non-degenerate part) of X bases, and a 3' z-fold degenerate end of Y bases. CHX + Y-z + 2GT: CodeHop primer designed at the intron limit, which contains the first two bases of the intron (by mistake, we reversed the two bases in the single such case, i8F). DX-Y-mz-t: Classical degenerate primer of X bases long, Y-fold degenerate, containing z to t mismatches according to the species (despite degeneracy in primer design). NHX+Y-Z: We called this a 'Nucleotide-hop' primer, by homology with CodeHop primers, but design was based on nucleotide alignment; we designed a 5' clamp (non-degenerate) and degenerate the 3' end according to the set of nucleotide sequences available thus ignoring codons. NHX+Y-Z-mz-t: Same as above, but, despite primer degeneracy, there may remain mismatches in some species; in this case there are from z to t mismatches according to the species for which we have sequence data. For instance, a primer (D30-1-m0-2) actually does not contain ambiguity bases (-1: not degenerate), and contains 0 to 2 mismatches according to the species. Other symbols: * this primer was not used. # erroneous primer sequence, the subsequently corrected primer i13Rcor was not tried

**Figure 2:**
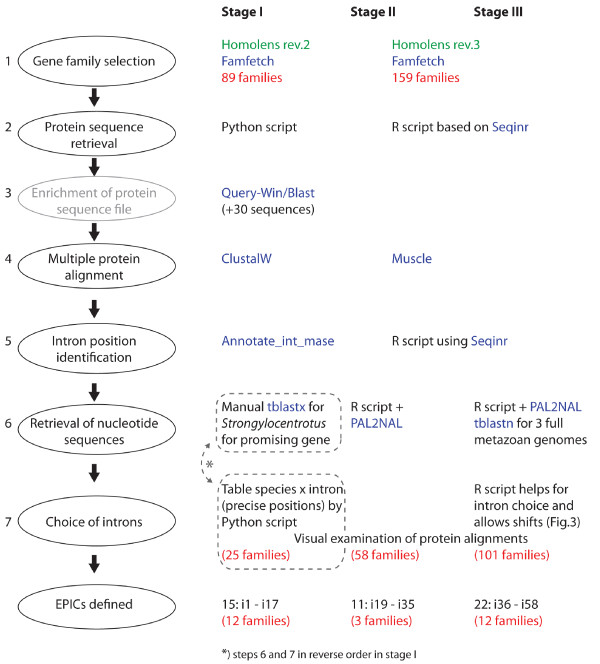
**Flowchart representing the different steps and stages of the bioinformatic assessment of EPIC loci**. Step 3 was not performed in stages II and III. Steps 1-5 were identical for stages II and III. Visual examination of protein alignment (part of step 7) was performed for all stages I-III.

**Figure 3:**
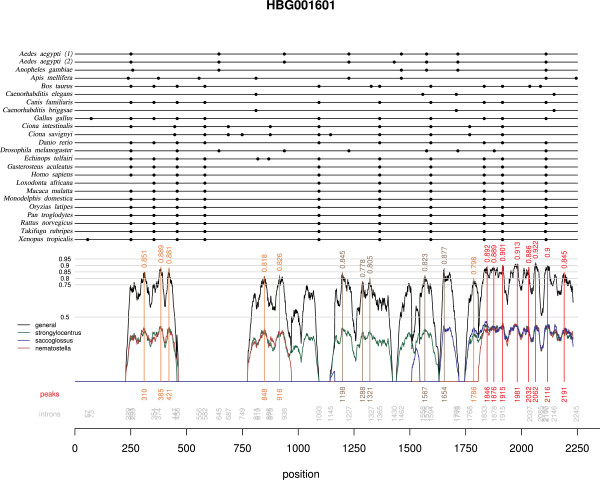
**Example of a graphical representation (stage III) of the multiple nucleotide alignment**. This tool was introduced at stage III, to help select conserved regions encompassing introns for PCR primer design. The multiple alignment of the gene family retrieved from *Homolens *appears at the top; dots indicate intron occurrence (intron positions are reported in gray at the bottom of the graphic). The similarity score ω (black), as well as similarity scores with *Strongylocentrotus *(ω_1_, green), *Saccoglossus *(ω_2_, blue), and *Nematostella *(ω_3_, red) are plotted at the bottom of the graphics; for a better readability, ω_1 _... ω_3, _are halved. Peak of nucleotide conservation and corresponding ω values and positions on the multiple alignment are identified by vertical lines (a colour code indicates the number of species for which additional sequences were available).

The number of introns for which we designed and tested primer pairs was respectively 15 (named between i1 to i17), 11 (i19 to i35) and 22 (i36 to i58) for stages I, II and III described in the Methods section (Fig. 1, Fig. 2). Intron numbers are not consecutive because we could not design satisfactory primer pairs for some introns that appeared good at the preceding step. In addition, we designed primers for two genes containing already known "universal" EPICs: in *ATPS-α*, one intron corresponds to the one in Jarman et al (2002) [11] with slightly different primer sequences, and primers were designed for an additional intron; in *EF1-α*, two primer pairs were designed for each of two introns. The 52 introns came from 29 different gene families and corresponded to 93 primer pairs tested in all species. Surprisingly, a single family was retained from both stages I and II (so that intron 2 is the same as intron 22). From the 89 families containing no duplication nodes (i.e. a node containing the same clusters of taxa several times, due to gene duplication) isolated during stage I, only six introns appeared to be present at exactly the same nucleotide position in 100% of the species, and for only 3 of these, corresponding to EPIC 1, 4 and 5, could we design satisfactory primer pairs (Fig. 1). Thus, we also designed primers for some introns that were present in 100% of the deuterostome species in the amino-acid alignments (i.e. vertebrates and *Ciona*, no echinoderm species being available in the *Homolens *database). For subsequent stages, intron position was not necessarily perfectly invariant (see Methods). Fig. 3 displays an example of the output of the graphical script designed for stage III. For 36 introns (56 primer pairs out of 93) we could define a forward and a reverse primer whose degeneracy did not exceed 8-fold. For 12 of them (22 primer pairs), degeneracy was lower than or equal to 6. In particular, the primers designed for introns 21, 25, 26, 45, 50 and 54 displayed no more than a 2- or 4-fold degeneracy (Fig. 1).

### Effect of taxonomy on PCR results

**Figure 4:**
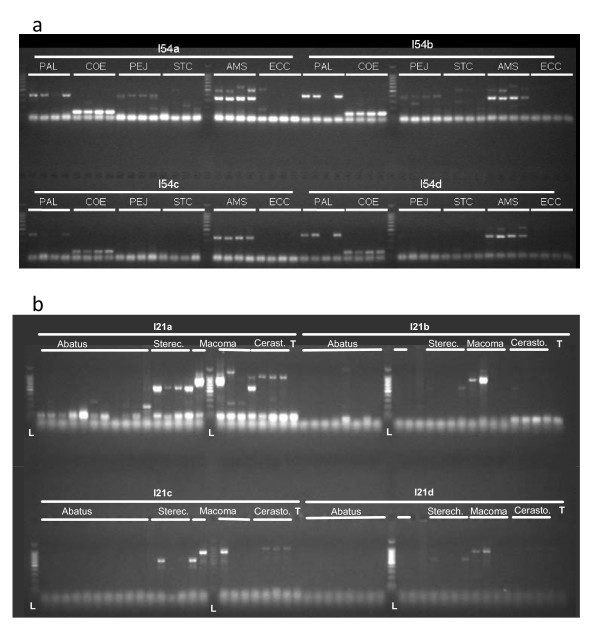
**Agarose gel electrophoresis results for 4 primer pairs (one 96-well PCR plate)**. a: intron 54 (standard protocol); b: intron 21 (S-CR protocol). The size marker, labelled L, is a 100 bp ladder with the brightest band corresponding to 500 bp. (a) Four individuals of each species are presented, in the following order: *P. lividus, C. eumyota, P. japonica, S. clava, A. squamata, E. cordatum*, for intron 54 (primer pairs: a, b, c and d). (b) Lanes successively correspond to the following species (number of individuals in parentheses): *Abatus cavernosus *(4), *A. agassizi *(3), *A. cordatus *(3), *A. nimrodi *(1), *Sterechinus neumayeri *(3), *S. agassizi *(1), *Macoma balthica *(4), *Cerastoderma edule *(4).

For each stage, protein family, and intron, Table 1 and Table 2 report the results of the PCR for each genus as observed on agarose gels (Fig. 4) (and the total number of genera), distinguishing the three quality levels 'P'(Promising), 'I' (Intron-size amplicon) and 'A' (Amplification) defined in the Methods section V. The number of introns producing an amplification product large enough to contain an intron of at least 70 bp, i.e. pooling the 'P' + 'I' categories, is rather similar among genera, and varies from 20 to 30 introns (out of 51 tested), i.e. from about 40% to 60% of the introns. Some of the factors likely to generate variation in this value among taxa are: (i) the number of PCR conditions tested among genera, (ii) DNA extract quality, (iii) phylogenetic distance of the taxon from *Strongylocentrotus *and *Ciona*, the species which most influenced primer design, (iv) the molecular evolutionary rate of the phylum or lineage, and (v) the average size of introns in the taxon (some taxa tend to have large, thus not amplifiable introns, or too small introns eventually recorded as 'A' despite the presence of an intron). The number of "promising" introns ('P') appears more variable among taxa (from 6 to 15 introns, i.e. 12% to 29%). Factors causing variation in the proportion of "promising" introns (and which should not necessarily affect the number of 'P+I' introns) may be: (i) quality of DNA extracts (see below, section 5) since null PCR may infrequently occur due to bad DNA extracts and cause a locus in a genus be scored as 'I' instead of 'P' (even if a single individual of the 4 extracts tested does not amplify), (ii) variable tendency to display null alleles (e.g. alleles producing no amplification products) among taxa (a consequence of intrinsic polymorphism or effective population sizes), and (iii) genomic features such as the ploidy level or the frequency of transposable elements (causing multiple band patterns, for instance).

**Table 1:** Results of PCR amplification for each intron locus in each genus.

Intron	1	2	3	4	5	6	7	8	9	11	12	13	14
Homolens version	II	II	II	II	II	II	II	II	II	II	II	II	II
Gene family (HBG-code)	004117	001043	003635	008731	052978	001266	006832	008594	052978	040291	040291	006550	005089

No primer pairs tested (without errors)	2	1	1	1	2	1	1	1	1	1	1	1	1

PCR size expected if intron absent	174	100	80	?	90-130	75	60	110	90	70	150	170	110

No genera 'P'	6	8	0	0	8	0	0	0	3	1	1	0	0
No genera 'I'	3	2	4	0	2	0	1	4	5	7	7	1	1
No genera 'A'	1	0	0	0	0	0	0	0	1	0	0	0	0

*Paracentrotus*	I	P			P				P	I	I		
*Amphipholis*	P	P	I		P		I		P	I			I
*Echinocardium *(all conditions)	P	P	I		P					I	P		
*Corella*	A	P	I		P				I	I	I		
*Perophora*	P	P			P				I	P			
*Styela*	P	P			P				P	I	I		
*Abatus *(all species)	I	P			P			I	I		I	I	
*Sterechinus *(all species)	P	P			I			I	I		I		
*Macoma*	I	I	I		I			I	I	I	I		
*Cerastoderma*	P	I			P			I	A	I	I		
*Corallium*	P	I	na	na	I	na	na	na	I	na	na	na	na
*Paramuricea*		na	na	na	I	na	na	na	na	na	na	na	na

**Intron**	**15**	**17**	**19**	**21**	**22**	**24**	**25**	**26**	**29**	**30**	**34**	**35**	**36**

Homolens version	II	II	III	III	III	III	III	III	III	III	III	III	III
Gene family (HBG-code)	000163	003635	000144	001043	001043	001043	001601	001601	002428	002428	002428	002428	005337

No primer pairs tested (without errors)	1	1	2	4	1	2	4	4	1	1	2	4	2

PCR size expected if intron absent	170	135	120-135	120	150	180	193-210	180	165	155	155-165	120-155	125-145

No genera 'P'	3	0	2	5	2	0	5	0	5	2	3	0	4
No genera 'I'	6	0	0	5	3	0	5	1	3	2	2	0	4
No genera 'A'	1	0	4	0	3	0	0	0	0	6	1	0	0

*Paracentrotus*	I		P	P	P		I		P	P	P		
*Amphipholis*	I			I	I		I		I	A			I
*Echinocardium *(all conditions)	I			P	P		I		P	I			
*Corella*	I			I	I		P		I	A			I
*Perophora*	P		P	P			P			A			P
*Styela*	I			I			P		I	I	A		P
*Abatus *(all apecies)	P		A	P	A		I		P	P	P		P
*Sterechinus *(all species)	A		A	P	I		P		P	A	I		I
*Macoma*	I		A	I	A		P	I	P	A	I		I
*Cerastoderma*	P		A	I	A		I			A	P		P
*Corallium*	I	na	na	I	I	na	P	na	I	na	na	na	
*Paramuricea*	I	na	na	na	na	na	na	na	na	na	na	na	

**Intron**	**37**	**38**	**39**	**40**	**41**	**42**	**43**	**44**	**45**	**46**	**47**	**48**	**49**

Homolens version	III	III	III	III	III	III	III	III	III	III	III	III	III
Gene family (HBG-code)	007493	032846	032846	010911	010054	009263	039475	015649	015649	026608	026608	011376	011376

No primer pairs tested (without errors)	1	1	1	1	2	1	4	1	1	1	1	1	4

PCR size expected if intron absent	125	160	95	180	255	140	110-145	135	150	75	215	285	240-260

No genera 'P'	4	1	2	0	0	0	5	1	1	1	0	4	0
No genera 'I'	2	2	0	0	2	4	2	1	4	3	1	5	0
No genera 'A'	0	1	0	0	0	0	0	3	0	0	2	0	0

*Paracentrotus*		A	P		I			A		I			
*Amphipholis*	I	I				I	I	I		I	I	P	
*Echinocardium *(all conditions)	P		P			I	P	A				I	
*Corella*						I	P	P	I			I	
*Perophora*	P	I					P	A	P	I	A	I	
*Styela*	P	P				I	I		I		A	P	
*Abatus *(all species)	I				I		P			P		P	
*Sterechinus *(all species)												P	
*Macoma*							P		I			I	
*Cerastoderma*	P								I			I	
*Corallium*	A	I			na		P	na	I	P	I		A
*Paramuricea*	I	I			na		I	na		I	I		

**Intron**	**50**	**51**	**52**	**53**	**54**	**55**	**56**	**57**	**58**	**ATPsαJ**	**ATPSαi2**	**EF3**	**EF4**

Homolens version	III	III	III	III	III	III	III	III	III				
Gene family (HBG-code)	011376	011376	031768	031768	031768	031768	031768	007173	007173				

No primer pairs tested (without errors)	1	4	1	2	4	1	2	1	1	1	1	4	4

PCR size expected if intron absent	120	240	260	120	75	160	260	145	210	90	330	250-280	210-35

No genera 'P'	6	5	1	5	3	1	2	2	0	2	2	1	3
No genera 'I'	4	4	0	2	4	1	2	1	1	4	3	4	6
No genera 'A'	0	0	0	0	1	0	0	0	0	0	0	4	1

*Paracentrotus*	P	P	P	P	P			I				A	I
*Amphipholis*	P	I		I	P					I	I	I	I
*Echinocardium *(all conditions)	I			P	P	P				I	I		I
*Corella*	P	I			A		P			I		I	A
*Perophora*	P	P			I		I			I		P	I
*Styela*	I	I			I		I			P		A	I
*Abatus *(all species)	I	P		P	I	I		P			I	I	I
*Sterechinus *(all species)	P	P		P	I			P			P	I	P
*Macoma*	P	I		I					I	P		A	P
*Cerastoderma*	I	P		P			P				P	A	P
*Corallium*	I	P	A	P	I	A	A		na	na	na	na	na
*Paramuricea*	P		na	P	I		P	P	na	na	na	na	na

Empty cells correspond to absence of amplification product or occurrence of primer dimer; "na" (not available) corresponds to introns not tested in the considered genus

**Table 2:** Summary of results for each genus.

Total introns	P	I	A	Nulls	Total
*Paracentrotus*	15	9	3	25	52
*Amphipholis*	7	23	1	21	52
*Echinocardium *(all conditions)	13	11	1	27	52
*Corella*	7	15	3	27	52
*Perophora*	15	8	3	26	52
*Styela*	10	14	3	25	52
*Abatus *(all species)	14	14	2	22	52
*Sterechinus *(all species)	12	9	3	28	52
*Macoma*	6	18	4	24	52
*Cerastoderma*	11	9	5	27	52
*Corallium*	6*	12*	5*	6*	29
*Paramuricea*	4*	8*	0*	10*	22

Total number of introns in each category of results. *: cnidarians were not tested for all introns.

**Figure 5:**
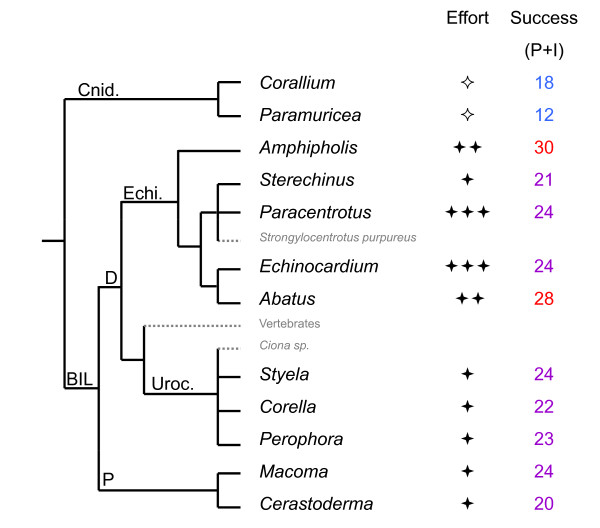
**Phylogenetic relationships **[39]**among the genera tested and global results for intron amplification**. To the right of the tested genus (name in black) symbols reflect the level of technical effort [✧ Not all primer pairs were tested, ✦ standard effort, ✦✦ more tests than standard (either PCR conditions or DNA extracts), ✦✦✦ several of the previous improvements], and the success column gives the number of introns scored as 'P' or 'I'. The taxa whose sequences or genomes most influenced primer design, either by being our models for the non-degenerate part of the codehop primers or by over-representation in gene family databases, are written in small grey letters. Major phylogenetic splits are indicated using the following abbreviations: BIL (Bilateria), P (Protostomia) D (Deuterostomia), Echi. (phylum Echinodermata), Uroc. (phylum Urochordata), Cnid. (phylum Cnidaria).

Surprisingly (Fig. 5) we did not observe any clear relationship between phylogeny and global PCR success (i.e. number of successful loci per genus, whatever the quality level considered as successful), with an average of 22, 23 and 25 introns amplifying (I or P) per genus in bivalves, ascidians and echinoderms respectively (about 10-12 of which yielded promising 'P' patterns in each group). Some genera belonging to protostome phyla displayed better results than some deuterostomes. The cnidarian *Corallium*, in which only 29 loci instead of 52 were surveyed, provided 18 amplifying introns (I + P). This absence of detectable phylogenetic influence is probably not an artefact resulting from variation in technical effort since, even when considering only the six initial species of the standard protocol (Table 3) that were tested under a larger range of PCR conditions, the highest variation occurred within phylum (Table 2). Urochordates and molluscs, for which we applied the same level of technical effort, display very similar amplification results despite a very different phylogenetic distance relative to species which most influenced primer design. Despite testing fewer primer pairs on the cnidarians, they display only slightly lower numbers of "P+I" intron loci than other taxa. Ascidians tend to display small introns (data not shown, available on request), consequently cases of null amplifications due to excessively large fragment size are expected to be less numerous, though this may also lead to reporting the absence of an intron if it is too small ('A' result instead of an 'I or P'). For example, *Corella eumyota *displays a lower number of successful loci and smaller intron sizes than other ascidians.

**Table 3:** The different protocols and the species and loci (primer pairs) to which they were applied.

Protocol name	DNA extraction	PCR program	Primer pairs tested	Species
Standard	Phenol-chloroform (except *P. lividus *stage III (i21-i58) where we used Promega extracts)	TD6	All	Pl, As, Ec, Ce, Sc, Pj (4 ind. per species)
S-F	Idem	Std-Fix	1, 2, 3, 4, 5, 6, 7, 9, 13, 14, 15, 17	Pl, As, Ec, Ce, Sc, Pj (4 ind. per species)
S-F-60	Idem	Fix50-60	5b, 21a-d, 25a-d, 35ab, 19ab, 22, 24ab, 29, 30, 34ab, 49a-d, ATPSαJ, ATPSαi2	Pl, As, Ec, Ce, Sc, Pj (4 ind. per species)
S-CR	According to taxon and individuals	TD6'	All	Aca(4), Aa(3), Ac(3), An(1), Sn(3), Sa(1), Mb(4), Ce(4)
EE4	1 Qiagen + 1 Promega + 1 Chelex + 1 CTAB-phenol	EE-GP	All	Ec(4)
EE16	8 Chelex + 8 Qiagen	EE-GP	1b, 5b, 22, 25, 29, 37, 43, 53b, 54c, 55, ATP-Sa, EF4c	Ec(16)
GP	8 Promega	EE-GP	All introns, but only with the primer pair "a" (Fig. 1)	Pl(8)
DA	3 Qiagen + 1 Phenol-chloroform	DA	All primer pairs tested for 29 loci in Cr, 22 in Pc (intron numbers in Table 1)	Cr(3-4), Pc(3-6)

The letters a, b, c, and d after the intron number refer to primers F and R, F2 and R, R2 and F, F2 and R2 respectively. DA, EE, GP and CR refer to initials of the authors who used them. ind.: individuals.

**Figure 6:**
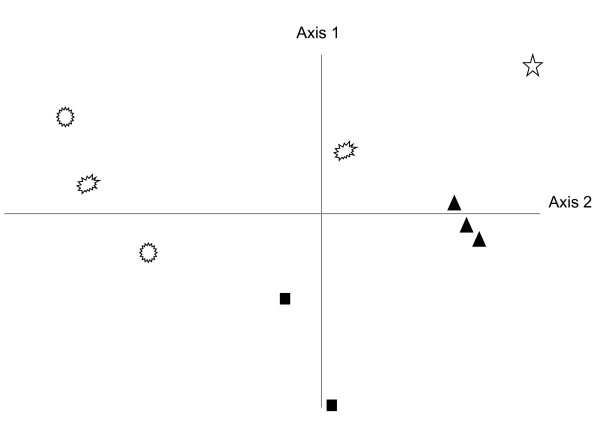
**Correspondence analysis representing the bilaterian genera according to their results for each intron**. Nulls, A, I and P were respectively scored as 0,1,2 and 3. Methodological changes such as (i) adding cnidarians (therefore reducing the number of variates from 52 to 22 or 29), (ii) considering nulls (scored as 0) versus all amplifying categories (1) or (iii) changing the nature of the multivariate analysis did not change the pattern. When included in analyses, the two cnidarians appeared neither to form a tight group, nor to be outliers relative to bilaterian phyla. Empty symbols represent genera of the Echinodermata (stars, circles and ovoids intuitively represent ophiuroids, regular sea uchins and irregular sea urchins, respectively), black triangles represent ascidians (Urochordata) and black squares represent the two bilvalves (Mollusca).

The absence of a relationship between global success and phylogenetic position in bilaterians is probably explained by the rules we used to select intron loci before primer design. In fact, the zones selected for the 3' region of the primer design were generally invariant in their amino-acid sequences. Therefore, a good predictor for primer matching may be the similarity of the genome nucleotide composition (influencing codon preference) to the nucleotide composition of the reference species (*Ciona, Strongylocentrotus...*); since nucleotide composition is variable even at low taxonomic levels (e.g. [20]), the absence of phylogenetic effect is not surprising. The only source of variation is therefore the degeneracy of the code; this is known to vary greatly even within species, so focusing on phylum-specific primers may not be useful when very few taxa are available for a phylum. Actually we have now cloned and sequenced some of these EPICs in echinoderms and we nearly always observe variation within species (even within populations) in the exon sequence (synonymous changes). However, when the precise pattern at each intron was considered and the data (from Table 1) were analysed through a factorial correspondence analysis, the genera seemed to group according to their phylum (Fig. 6, see legend for details), illustrating the fact that some introns are more useful in some phyla than in others. Within echinoderms, more genera were surveyed, but we did not observe any taxonomic trend, either comparing ophiuroids *versus *echinoids, or regular *versus *irregular echinoids. Echinoderms appear widely scattered, by contrast with Urochordates. This may reflect different genome evolutionary rates of those phyla though a richer taxonomic sampling is required to test this hypothesis.

Some gene families appear to be extremely good providers of EPIC markers (Table 4). Remarkably, five introns, from four different gene families, amplify intron-sized products ('P' or 'I') in all the metazoan genera tested (ten to twelve depending on intron) (Table 4). The previously known universal EPICs tested, ATPS and EF1, do not belong to these families.

**Table 4:** Gene families providing best introns and highest numbers of introns.

Gene Family	Gene name (from *Homolens*)	EPIC n°	No. bilaterian genera "P or I"	CR	PC
HBG0011376	UDP-N-acetylglucosaminyl-	48	9	NA	NA
(*Homolens *3)	transferase	49	0	0	NA
		**50**	**10**	**1**	**1**
		51	9	1	NA

HBG0052978	Ubiquitin	**5**	**10**	**1**	**1**
(*Homolens *2)		9	8	1	NA

HBG004117	Calpain	**2 = 22**	**10**	**1**	NA
(*Homolens *2)		**21**	**10**	**1**	NA
= HBG001043					
*(Homolens 3*)					

HBG0001601	LD39850p; Peptidylprolyl	**25**	**10**	**1**	**1**
(*Homolens *3)	isomerase domain and WD				
	repeat-containing protein 1				

HBG0031768	Glutamyl-prolyl-tRNA-	52	1	0	NA
(*Homolens *3)	synthetase	53	7	1	1
		54	8	1	1
		55	2	0	NA
		56	4	0	1

HBG0002428	AT23778p; Colon RCB-0549 Cle-	29	8	1	NA
(*Homolens *3)	H3 cDNA; RIKEN full-length	30	4	NA	NA
	enriched library; clone: G430060P17 product:si	34	5	NA	NA

The ten bilaterian genera were tested for all 52 introns, two cnidarian genera were tested for 22 and 29 introns respectively. The number of bilaterian genera displaying promising or intron amplifying patterns (P or I) out of the ten genera tested is indicated. The five best introns (10/10 success among bilaterian genera) appear in bold. In the right-hand columns, the results of the two cnidarian genera when tested (not all loci): NA (not available), 1 (promising or intron amplifying), 0 (no intron-size amplicon).CR: *Corallium rubrum*, PC: *Paramuricea clavata*.

### Effect of primer design

**Figure 7:**
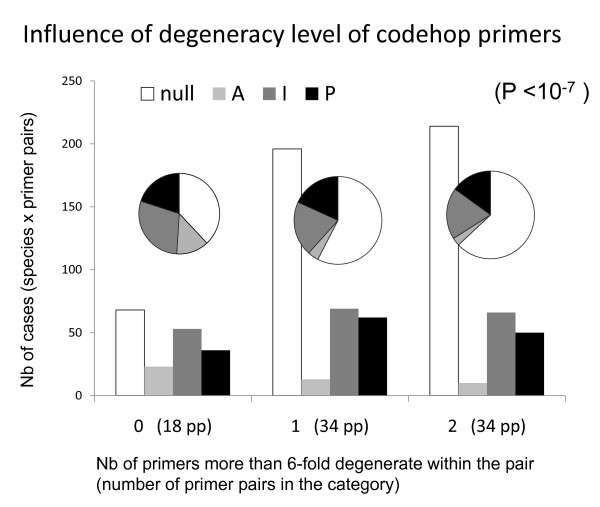
**Effect of the degeneracy levels of codehop primers on PCR results**. Results from the 10 bilaterian genera in which both primers were "codehop" were considered here (they cannot be directly derived from Table 1 since, for some introns, not all primer pairs were "codehop"). The bars represent the total numbers of cases (species × primer pairs) displaying results 'P', 'I' 'A' or nulls, for three categories of introns, those in which no (0), one (1) or both (2) primers have a more than 6-fold degeneracy. Exact tests were performed from the 4 × 3 contingency table used to build the histogram, as well as from tables derived from it after pooling some columns (for instance nulls versus "A + I + P"): all were highly significant. The pie chart diagrams display the proportion of the four categories of results within each category of degeneracy; they illustrate the increase in the proportion of null loci when primer degeneracy increases.

The least degenerate primers provided the best results (Fig. 7, highly significant exact tests) for the primer pairs composed of two codehop primers (86 cases). This result was not trivial: if the taxa available in our protein alignments had not been sufficiently representative of the diversity of phyla tested--among deuterostomes, for instance, the *Homolens *database provided only vertebrates and one urochordate genus--higher amplification success would have been observed for more degenerate primers. Despite this significant relationship, some of the least degenerate introns at the stage of primer design never amplified (e.g. introns 6, 7, 24 and 49). Codehop primers seem slightly more efficient than primers designed from nucleotide alignments only, though this is not statistically significant (additional file 1).

### Effects of PCR program and extraction method

Comparisons, for 12 primer pairs, of touch-down and fixed annealing temperature programs suggest that touch-down programs are more stringent (less success) and less prone to produce artefactual additional fragments (higher proportion of 'P' patterns) (additional file 1). The two-phase program (tested on 28 primer pairs) also appears to help (additional file 1). However, we obtained no statistical support for these effects. Since in most experiments we used program TD6, our global results may be improved by using alternative programs.

A strong influence of DNA extraction and/or tissue storage history, depending on the species, was revealed (additional file 1).

### Obtaining new EPIC loci for any eukaryote lineage

Our method can potentially be applied to obtain new EPIC loci for any phylogenetic group. Three strategies can be followed. (i) Using the last version of *Homolens*, the famfetch tool, and the graphical tool developed in this study, one can isolate numerous new EPIC loci, eventually decreasing the stringency level allowing duplication nodes in some genomes, if numerous loci are desired. (ii) More simply, one can focus on the gene families we isolated, and retrieve from protein, nucleotide or EST sequence databases the entries corresponding to the desired lineages; primer pairs should then be designed following our method. (iii) One can just test the primer pairs developed in our study (Fig. 1), preferably using several DNA extraction methods, which can be done in less than 10 working days and for several species simultaneously. Even for kingdoms very distant from metazoans (e.g. plants, fungi, or protists) the second approach may provide successful loci since in some of these gene families the protein alignment files included sequences from plants. We can predict that the success rate will not depend much on the phylogenetic proximity of the taxa to deuterostomes (since those protein sequences are definitely strongly conserved) but will be influenced more on average by the genomic substitution rate of the taxonomic group. For instance, the ecdysozoan phyla (e.g. arthropods and nematodes) may not work as well as molluscs and annelids, though these phyla share a common ancestor relative to deuterostomes, since their genomes are known to evolve rapidly at the nucleotide level [21,22]. For the third strategy, based on already defined primers, genome nucleotide composition may be an important factor (see above).

The facts that (i) low degeneracy codehop primers performed better than high degeneracy primers, and (ii) phylogenetic distance (to vertebrates and *Ciona*) has no relationship with global amplification results, are positive experimental findings of this study, and suggest that the simplest strategy (use of the primers we defined) may be sufficient in most metazoan species to obtain several EPIC loci. In the increasing number of phylogenetic groups where sufficient EST sequence data are available to enrich nucleotide alignments in a variety of taxa, primer design may not require using the Codehop strategy (personal observation from a new ongoing EPIC project).

### From EPIC identification to genotyping of large samples

Once a promising EPIC locus has been found, it is not always straightforward to directly characterize populations with it. Obtaining sequence data prior to genotyping is recommended. In some cases, good results are provided by direct sequencing of PCR products. There are generally no indels in the exon sequence, so the sequences are readable even with ambiguous positions due to heterozygosity in this region, allowing the design of specific primers. In some (*e.g*. inbred) species where homozygous individuals are common, direct sequencing is very useful, and eventually, "heterozygous sequences" may be deduced automatically from sequence files containing ambiguities, using dedicated software [23,24]. When direct sequencing is not satisfactory, we recommend cloning the EPIC amplicons from a dozen individuals and sequencing about 10 clones per individual to assess the nature and the level of the variation, permitting (i) the definition of more specific primers if necessary and (ii) the decision whether to characterize alleles by sequence, by size, or by conformation. Some of these introns have been cloned and sequenced in large samples of *P. lividus*, *E. cordatum *and species not included in the present study, and in all cases polymorphism is high due to both indels and substitutions (unpublished). In some cases, the EPIC locus provides diploid Mendelian variation in fragment size visible on agarose gels. Alternatively, finer variations can be revealed using PAGE or automatic sequencers [1]. Conformation techniques (SSCP, DGGE or, more recently, melting curve genotyping (e.g. [25]) allow determination of allele classes, but they have a sensitivity limited to relatively small fragments and they provide no information on allele relationships. The richest information for diploid specimens is a diploid sequence genotype, but classical sequencing techniques do not deal well with heterozygosity. "Next generation sequencing" technologies, such as "454 sequencing", in addition to their extreme rapidity, should allow sequencing a mixture of numerous PCR products, such as for instance from 15 EPIC loci in each of 50 or more individuals, identified by individual labels (4 base sequences, inserted in PCR primers or linked to PCR products) for reasonable prices (less than 2000 € in 2009). These approaches differ in throughput, cost and sensitivity (Table 5).

**Table 5:** Benefits and limits of different genotyping techniques for EPICs (for direct sequencing, see Results-Discussion, last section).

Genotyping technique	454 sequencing	Cloning-sequencing	Melting curve genotyping	SSCP	Intron length polymorphism
Information (models of allele evolution)	Diploid Seq. (various)	Diploid Seq. (various)	Genotype (IAM)^i^	Genotype (IAM)^i^	Genotype (IAM)^i^
Fragment size	Seq <400 bp*	No limit **	Best <350 bp	Best <350 bp	No limit
Allele number	No limit	No limit	Very limited. If high, impairs genotyping	Some alleles may not be distinguished	Some alleles may not be distinguished
Throughput	high	low	high	Medium	medium
PCR	Classical	Classical	Real Time	Classical	Classical
Cloning	No	Yes	No	No	No
Pooling loci	After careful quantification	Possible^# ^but problematical	No	Possible	Common
Electrophoresis	No	For sequencing	No	Yes	Yes
Sequencing	1 run = Numerous individuals × numerous loci	Several reactions required per individual	No	No	No
Number human action steps ^μ^	L + 1 + 1	L + NL + 10NL	L	L + L/3 ^μ'^	L + L/3 ^μ'^

Seq.: sequence. ^i^: IAM: Infinite Allele Model (evolutionary distance is considered identical between all allele pairs). *: Larger amplicons can be included in the run, but only the first 400 bp will be sequenced; however, to favor equal representation among loci, amplicons of relatively uniform lengths (500-900) are preferred. Sequencing can be oriented if desired (start with one of the PCR primers only). This size threshold may increase in future. **: but cloning efficiency may depend on size (and size must be compatible with PCR). #: not recommended, since loci may be very unequally inserted by cloning vectors (often one missing). ^μ ^: For each technique, the number of steps requiring human action is expressed as a function of the numbers of loci (L) and of genotyped individuals (N) and deduced from the five preceeding rows of the table. We do not consider the possibility of multiplex PCR (i.e. several loci in a single PCR reaction) though this reduces the number of steps). ^μ'^: Pooling three loci together for electrophoresis is generally easy (whether or not an automatic sequencer is used); more loci can be pooled when allelic distributions do not overlap.

## Conclusions

Our new method appears very efficient for finding universal intron loci (EPIC) in sequence data bases whatever the phylum, in metazoans. These EPICs, in addition to providing a set of independent nuclear markers for population genetics and phylogeography, can complement or replace the barcoding molecule used for metazoans, COI, resolving problems associated with single marker studies or inherent in the mitochondrial genome, such as its lack of variability in some phyla [26]. For about ten of these EPICs we obtained sequence data for several individuals, from one to nine distinct genera (unpublished data, available on request). Insertions and deletions were frequent (among which there were a few microsatellites), in only two cases there was no polymorphism, and a minority of cases displayed paralogs or distinct groups of sequences, a problem which was solved when internal primers were defined. This study therefore fills a serious gap in the toolbox of molecular ecologists. These new and universal EPIC loci should generate multilocus sequence datasets from populations of numerous non-model species. Relative to single-locus sequence data sets or multilocus microsatellite loci datasets, the inferences made from such data using the coalescent theory will be much more precise.

## Methods

### Bioinformatic assessment of candidate loci

We had previously tried a method based on the Exon-Intron Database [26,27,8] but this was not successful and thus we developed an original approach. In order to find universal markers from orthologous genes, we took advantage of annotated full-genome sequences to obtain a list of candidate gene families.

The search involved three successive stages (I-III), introducing some methodological changes between stages; each stage contained the following steps, except that step 3 was not performed for stages II-III (see Fig. 2 for flowchart).

#### Step 1: Gene family selection

The *Famfetch *software [28] was used to query revision 2 (stage I of our search) or revision 3 (stages II and III) of the *Homolens *database of homologous gene families [29] from *Ensembl*, and retain families for which orthologs were found in *Homo*, Rodentia, *Canis, Gallus, Danio, Xenopus*, Percomorpha, *Ciona*, Insecta, and Nematoda, but no duplication event occurred at the root of any of these taxa in the family tree. This step eliminated known highly conserved families, including most or all of the already known "universal" EPIC loci, but we estimated that the remaining loci would be less prone to paralogy and laboratory effort would be saved. Families in which one isolated species within one of these taxa appeared duplicated were not eliminated (e.g. family HBG001601 was retained (Fig. 3) although *Aedes aegyptii *presents a duplication, since the duplication is not shared by all Insecta). Step 1 provided 89 gene families for stage I, and 159 gene families for stages II and III, which were merged until step 6.

#### Step 2: Protein sequence retrieval

For each retained gene family, protein sequences were retrieved with a python script, for stage I, or an R script based on the *Seqinr *package [30], for stages II and III.

#### Step 3: Enrichment of protein sequence file

For stage I only, we enriched our protein alignment with protein sequences from other deuterostome species using the Query-Win software to obtain Genbank accession numbers for CDS of deuterostome taxa that were not already included in Homolens. For each gene family, blast searches using the *Homo *and nematode sequences were performed on this new database; sequences leading to a lower than 10^-100 ^e-value were considered as homologous sequences. Only 30 additional sequences were identified by this additional step.

#### Step 4: Multiple-sequence protein alignment

For each family, a multiple alignment of protein sequences was obtained with *ClustalW *[31] (stage I) or *Muscle *[32] (stages II and III).

#### Step 5: Intron position identification

Intron positions and phases were annotated on the multiple alignment using either the *Annote_int_mase *program (kindly provided by L. Duret, stage I) or an R script using *Seqinr *(stages II and III).

#### *Step 6 and 7*: Retrieval of nucleotide sequences, and choice of introns

Choice of introns occurred before retrieval of nucleotide sequences for stage I and after retrieval for stages II and III. For stage I, we first considered intron positions present in 100% of all species in all taxonomic groups (as determined by a python script). When such positions were considered promising (embedded within conserved and low-degeneracy amino-acid sequences), we manually retrieved corresponding nucleotide sequences using *tblastx*, for *Strongylocentrotus purpuratus *at least (and in some cases a few other species), to determine the 5' part of the codehop PCR primers (see primer design section).

Stage II was initiated before all stage I families were thoroughly visually screened since we estimated that the method changes introduced in Stage II would save time. For stages II and III, CDS were retrieved and the corresponding nucleotide alignment was obtained using *PAL2NAL*, for each family. The first 58 protein alignments, taken by increasing *Homolens *(HBGxxxxxx) number, were examined visually, looking for introns that were present in all the deuterostome species of the alignment and that lay between regions conserved enough to design PCR primers (see below).

In stage III, the remaining 101 families identified from *Homolens *revision 3 were examined: for this stage, we retrieved potentially homologous sequences from the unannotated genomes of *Saccoglossus*, *Nematostella *and *Strongylocentrotus*, by performing *tblastn *searches involving the protein sequences of *Homo *and *Ciona*. We retained sequences leading to a lower than 10^-5 ^*e*-value, and for which protein sequence similarity with some sequences in the original alignment was higher than 0.5. For this stage we developed a new R script facilitating the visual identification of both intron position and exon sequence conservation in a multiple alignment. This tool allowed us to consider introns whose position varied only slightly among phyla, which may therefore potentially provide EPIC markers (Fig.3). Region conservation was scored by computing a local similarity score ω in a sliding window along the multiple alignment. This similarity score is the geometric mean of up to four components ω_0 _... ω_3 _depending on the number *n *of species for which additional sequences were available (1 ≤ n ≤ 3), where ω_0 _is the mean local nucleotide similarity between pairs of sequences retrieved from *Homolens*, and ω_1_, ω_2 _and ω_3 _are the highest nucleotide identities between pairs of sequences involving one sequence from *Homolens *and one sequence from *Strongylocentrus*, *Saccoglossus *or *Nematostella *(when sequences from these species were available). We used a sliding window width of 20 nucleotides to compute ω, and identified local peaks of nucleotide similarity in sliding windows of 51 nucleotides width. A plot of nucleotide conservation and intron occurrence was used to visually select promising loci. Contrary to stage I, we did not eliminate potentially usable EPIC loci whose position may vary by a few nucleotides.

In addition to this original research among *Homolens *families, we specifically examined two genes containing already known "universal" EPIC loci, the Elongation Factor 1 *α *subunit and the ATP-Synthase *α *subunit [10,11]. We retrieved the corresponding metazoan nucleotide sequences from Genbank and aligned them with DIALIGN, specifying that the alignment contained both coding (exon) and non coding regions (introns). Then, we visually examined these alignment files to identify potential EPIC loci.

### Primer design

The great majority of primers were designed using the *Codehop *method [33,34], which is based on an amino-acid alignment. The 3' end of the primer (at least 11 bases) is degenerate in a way that represents all possible nucleotide combinations considering that there are usually several codons per amino-acid. The 5' end, called a clamp, is not degenerate, so that, although it may not anneal during the first two cycles, it should, in the following cycles, perfectly anneal to the primers incorporated and replicated during previous cycles. Such a strategy allows amplification of relatively variable regions, while limiting the problems inherent in highly degenerate primers. Primers were designed only when it was possible to limit degeneracy to less than 32× (except for a single primer that has 64× degeneracy) and most of them have 8× or less degeneracy (Fig. 1). This rule, aimed at reducing the risk of simultaneous amplification of paralogs, eliminated numerous potential loci. In practice, to visually identify conserved regions containing low-degeneracy codons, we used the *Bioedit *software and customized the amino-acid background color code to reflect the level of codon degeneracy. In some cases when the nucleotide alignment appeared much less variable than predicted from codon degeneracy, conventional degenerate primers were designed, based on the nucleotide alignment (i.e. ambiguities were tolerated along the primer). In these cases, we often decided to design the primer in two parts, with a 3' part of at least 11 bp degenerate enough to represent all observed nucleotide sequences in our alignment and a non-degenerate 5' part, mimicking the *Codehop *design strategy [35]. Below, we will refer to these primers as NucHop primers. All details of primer design and sequences are in Fig. 1. The letters a, b, c, and d after the intron number refer to primers pairs F and R, F2 and R, R2 and F, F2 and R2 respectively. The design of the 5' clamp of the primer was based on the *Strongylocentrotus *nucleotide sequence when available, otherwise on *Ciona*.

### Taxa tested

Within Bilateria, there are two important clades, the Protostomia, which contain the Lophotrochozoa (such as annelids and molluscs) and the Ecdysozoa (such as arthropods and nematodes), and the Deuterostomia (to which vertebrates, urochordates, and echinoderms belong) (Fig. 5). We first had decided to focus on Deuterostomia in which more sequence data are available, and to survey additional ascidian (Urochordata) species (*Corella eumyota, Perophora japonica*, and *Styela clava*) and echinoderm species (regular sea urchins*: Paracentrotus lividus*, *Sterechinus agassizi*, *Sterechinus neumayeri*; spatangoid sea urchins: *Echinocardium cordatum*, *Abatus agassizi, Abatus cavernosus*, *Abatus cordatus*, *Abatus ingens*, *Abatus nimrodi*; brittlestars: *Amphipholis squamata*). To check whether the EPIC loci selected would be exportable to more distant phyla, we also surveyed two mollusc species (Protostomia), *Cerastoderma edule *and *Macoma balthica *(both bivalves), and two extremely divergent, non-bilaterian species, the cnidarians *Corallium rubrum *(the red coral) and *Paramuricea clavata*. These cnidarians belong to the Octocorallia and are non-symbiotic. Initially, a first round of six species was systematically tested with four individuals per species and the "standard protocol": the three ascidian species, and three echinoderms: *P. lividus, E. cordatum*, and *A. squamata*. Then additional tests were made for these echinoderms and additional species. Table 3 reports the number of individuals, loci and species tested per genus, and the corresponding technical conditions.

### Molecular methods

DNA was extracted using phenol-chloroform methods for *Paracentrotus lividus *and the three ascidian species, plus *Echinocardium cordatum*, *Amphipholis squamata*, and *Abatus *spp. [36]; a saline method was used for *Sterechinus *spp. [37], and the Nucleon phytopure kit for some *Abatus*. In addition, specimens of *E. cordatum *were also extracted using Chelex [38], or with the QIAamp^® ^DNA Kit (Qiagen), and specimens of *P. lividus *were extracted using the Wizard^® ^SV Genomic DNA purification system (Promega). *Paramuricea clavata *DNA was extracted using the QIAamp^® ^kit, and we used standard phenol-chloroform extractions for *Corallium rubrum*. Polymerase Chain Reactions were performed in a 10 μl final volume, using 0.25 u of GoTaq^® ^Flexi-DNA Polymerase and green buffer (Promega), in 5 mM MgCl_2_, and 0.2 mM dNTP, with 10 pmol of each primer, and with 10 ng of template DNA, except for cnidarians for which template DNA probably exceeded 10 ng. PCR cycling temperature programs with fixed annealing temperatures (Table 6) were first tried for a few EPIC loci (trials at 48, 50, 55, or 60°C, according to locus referred as "St-fix" or "fix-50-60" in Table 6), and compared with two touch-down programs (TD6, TD6'); subsequently, touch-down programs were preferred and were used for all primer pairs for all EPIC loci except cnidarians (referred as the standard protocol, Table 3). For *E. cordatum *and *P. lividus*, all loci were additionally amplified with fixed annealing temperatures (named EE-GP in Table1). For cnidarians, a similar program was used at 45°C (DA in Table 6). PCR products were visualized by electophoresis using 5 μl of product on a 1X-TBE-1.5% agarose gel (260 ml) in a Biorad SubCell Model 192 Cell, gels of 25 × 25 cm and 4 combs of 51 wells, during ca 1 h migration at 120 V, using 2.5 to 5 μl of 100 bp Benchtop ladder ^® ^(Promega) as size marker.

**Table 6:** Description of PCR programs.

Program	Description
Std-Fix	3' 94°; 35 × [40" 94°C; 1' (45, 48, 50, or 55°C according to experiment), 2'72°C], 3'72°C
Fix50-60°C	3' 94°; 8 × [30" 94°C; 1' 50°C, 1'72°], 26 × [30" 94°C; 30"60°C; 1'72°C], 3'72°C
TD6	2'94°C; 14 × [1'94°C,1'58°C to 45°C (- 1°C/cycle), 1'72°C], 25 × [30"94°C, 45"58°C, 45"72°C], 3'72°C
TD6'	2'94°C; 14 × [1'94°C,1'58°Cto 45°C (- 1°C/cycle), 1'72°C], 25 × [40"94°C, 40"58°C, 1'72°C], 3'72°C
EE-GP	2' 94°; 35 × [20" 94°C; 1' 45°C; 1'72°]; 5' 72°C
DA	3' 94°C; 30 × [1' 94°C; 1' 45°C; 1'72°C]; 5' 72°C

DA, EE and GP refer to initials of the authors who used them. ': minute. ": second.

### Nomenclature used to characterize the results to allow comparison of methods

Results of PCR amplification as observed from agarose gels were characterized, at first on a per species basis, using the following rules. 'Promising' results ('P' in Table 1) refer to the observation in all individuals of the species of amplification products that were not too faint, did not display numerous fragments (rare cases with three bands were admitted, when one was constant) and were long enough to contain an intron of *ca *70-100 bp at least. The second category is designated 'I' (intron-size amplicon), and corresponds to amplicons of the size of an intron that were very faint or displayed multiple bands or failed to amplify in at least one individual. The third category, 'Amplification' (designated 'A'), corresponds to amplification products too short to contain a useful intron. Results in the subsequent tables and text are reported by genus after pooling all the protocols that were tried. For the species tested with several protocols (*P. lividus, E. cordatum*), and for the genera where several species were tested (i.e. *Abatus, Sterechinus*), the best result is reported.

## Abbreviations

CDS: Coding DNA Sequence; EPIC: Exon Primed Intron Crossing; PCR: Polymerase Chain Reaction; SSCP: Single Strand Conformation Polymorphism; DGGE: Denaturing Gradient Gel Electrophoresis; PAGE: Poly-Acrylamide Gel Electrophoresis.

## Authors' contributions

TH performed the majority of laboratory work, and designed primers. NB and SM developed all the dedicated bioinformatic tools. CR performed the laboratory work for several species; GP, EE, DA and KMJ tested all/numerous primer pairs for one species, and EE, in addition, compared DNA extraction methods. AC conceived and supervised the project, selected loci and designed primers, and wrote the article. Other authors provided DNA extracts. Some authors in addition improved the manuscript. All authors read and approved the final manuscript.

## Supplementary Material

Additional file 1 **Influence of molecular biology methods on EPIC amplification success**. It contains three paragraphs: (1) Are the codehop primers more efficient than primers designed from nucleotide alignments only? (2) Effect of PCR programs, and (3) Effect of DNA extraction and tissue conservation history. Click here for file
