# Supplementary material for: An efficient method to find potentially universal population genetic markers, applied to metazoans
Source: BMC Evol Biol. 2010 Sep 13;10:276. doi: 10.1186/1471-2148-10-276 (PMC2949868; doi:10.1186/1471-2148-10-276)
Supplement: Additional file 1 — Influence of molecular biology methods on EPIC amplification success. It contains three paragraphs: (1) Are the codehop primers more efficient than primers designed from nucleotide alignments only? (2) Effect of PCR programs, and (3) Effect of DNA extraction and tissue conservation history. [file 1471-2148-10-276-S1.DOC]

Influence of molecular biology methods on EPIC amplification success

(1) Are the codehop primers more efficient than primers designed from nucleotide alignments only?

For each of eight introns (i5, i21, i25, i26, i34, i35, i36, i56), we could compare several combinations of primers; in three cases, the combinations containing more Codehop primers provide a higher number of promising genera (‘P’), in one case they provide a lower number of promising genera and in four cases they perform equally. For the number of ‘P + I’ genera, the trend also slightly favours codehop primers but in both cases this is not statistically significant (Wilcoxon paired signed-rank tests). A test performed on the number of species (not genera) yielded a more pronounced trend in favour of codehop primers, but this was still not significant.

No significant correlation was observed between the number of ‘A’, ‘P’, ‘I’, or ‘P+I’ genera obtained and the total length of either the 5’ clamps (32 to 71 bp) or the degenerate 3’ ends (20 to 32 bp) of the primer pair.

(2) Effect of PCR programs

For 12 primer pairs (i1, i2, i3, i4, i5, i6, i7, i9, i13, i14, i15, i17) the six deuterostome species (genera) tested under the standard protocol (Tab. 1, Tab. 2) were also tested with multiple fixed annealing temperatures from the options 45, 48, 50 and 52°C. The mean number of species amplifying clearly appears higher with the fixed annealing temperatures (on average 2 species / locus) than the Touch down (TD) program (1 species / locus), but the number of promising species (removing multiband patterns and smears) appears similar (0.8 versus 0.6), suggesting that TD programs are more stringent and are less prone to produce artefactual additional fragments.

For 28 other primer pairs (5b, 19ab, 21abcd, 22, 24ab, 25abcd, 29, 30, 34ab, 35abcd, 49abcd, ATPSa and ATPS-J) an original two-phases program was also tested (annealing at 50°C for the early cycles, followed by 60°C for the later cycles, i.e. program Fix50-60 in Tab.1). To obtain amplification, for most introns both programs performed equally, but the two-phase program appeared better for 5 introns, whereas the touch-down TD6 program was better for a single intron. To obtain promising patterns, the two programs appeared comparable (4 introns versus 3 introns were better with the two-phase and TD6 programs, respectively).

(3) Effect of DNA extraction and tissue conservation history

The effect of the extraction method cannot be rigorously assessed since we did not test different DNA extracts from the same tissue sample. However, our results strongly suggest an influence of DNA extraction and/or tissue storage history.

In *Echinocardium cordatum*, in addition to the standard protocol (using phenol chloroform DNA extracts made specifically for this EPIC survey, from *a priori* well conserved tissues), all primer pairs were tested on DNA extracted using four different methods (using the EE protocol): the individual extracted with the Qiagen kit appeared promising for nine loci (25 primer pairs), the CTAB extract appeared promising for three loci (5 pp), the Promega kit extract gave one promising locus (1 pp) and the phenol-chloroform extract gave no amplification at all. The results obtained by the standard protocol (on all 93 primer pairs) with four individuals extracted using the Phenol-Chloroform method led to three promising loci only (5 pp). The simplest explanation is that the Qiagen DNA extract, due to better tissue storage or a better extraction method, was more easily amplified. The quality of DNA thus appears as a critical factor in amplification of EPIC loci. In addition, for 12 loci, eight individuals extracted with the Chelex method and eight individuals extracted with the Qiagen kit were tested using the EE protocol, and the total number of amplified individuals across all loci is 68 with Qiagen versus 27 with Chelex. When only promising patterns (‘P’) are considered, the difference decreases to 15 *versus* 12.

For the edible sea urchin, *P. lividus*, although the DNA extracts used for the standard protocol displayed very neat non-degraded bands in agarose control gels, only five loci appeared promising. The alternative protocol (GP), performed on DNA extracted with the Promega kit, yielded 14 promising loci, despite a single primer combination being tested per intron. Since the PCR program did not appear to have a strong influence on the number of promising loci, this result suggests that there is a (species-specific) influence of extraction methods on EPIC amplification success.
